# Reasons for Turnover Intention among Direct Care Workers in Korea’s Long-Term Care Insurance

**DOI:** 10.3390/healthcare8040395

**Published:** 2020-10-12

**Authors:** Yongho Chon, Yun-Young Kim

**Affiliations:** 1 Department of Social Welfare, Incheon National University, Incheon 22012, Korea; chamgil@inu.ac.kr; 2 Department of Social Welfare, Jeonbuk National University, Jeonju 54896, Korea

**Keywords:** long-term care, turnover, semi-structured interview, Korean LTCI system

## Abstract

This study explored reasons for turnover intention among direct care workers under the Korean long-term care insurance (LTCI) system. The author conducted semi-structured interviews with 19 care workers. The study revealed four main themes underlying the intention of care workers to change or leave their jobs. Care workers struggled with demanding working conditions, and their salaries were low. Moreover, the relationships with their directors and supervisors was not good, since some care workers felt that their directors despised them or gave them inappropriate instructions, and their supervisors did not complete administrative work fairly. Lastly, some workers’ health conditions prevented them from carrying out their care work. The results have implications for working practices of care workers, prices of LTCI services, training of directors and supervisors, and coverage of occupational health and safety insurance for care workers.

## 1. Introduction

Owing to the rapid population ageing and a decrease in caring roles being undertaken within families, care workers have become very important resources for older adults in need of long-term care (LTC) in many Western developed countries. However, it has been pointed out that many care workers experience heavy workloads, poor working conditions and income, and few opportunities for career development [1,2,3]. These issues have led to high turnover among care workers, some of whom have left the LTC field completely. Therefore, the recruitment and retention of care workers is a serious social challenge. Although many studies and policy measures have been implemented to tackle these issues, they persist in Western developed countries [4]. 

Similarly, some East Asian countries, such as South Korea (hereafter Korea), China and Japan have experienced challenges in recruiting and retaining care workers [4]. Specifically, in Korea, because of filial piety under the influence of Confucianism, it was considered a natural and even a moral duty for family members and relatives to take care of their aged parents [5]. Adult children felt a behavioral, physical, and financial duty to do so. However, this traditional care culture has been undermined by industrialization, urbanization, and individualism [5,6,7]. This has led to deficits in care for the elderly by their own family members and the socialization of care through the introduction of a new long-term care insurance (LTCI) system in 2008. However, although the new LTCI system was introduced only about ten years ago, many service providers have already experienced enormous difficulties in recruiting and retaining care workers [8,9,10,11]. The low pay and poor working conditions have been frequently found to be the main factors in the turnover of care workers [9]. Numerous (but piecemeal) measures have been implemented, and the challenges of turnover intentions of care workers persist in Korea [9,10]. The high turnover of care workers in the LTC setting is associated with costly recruitment and training of care providers and thus substandard care [2,12]. 

Care workers can be defined in many ways according to their roles and functions in each country’s care system. This study defines care workers as those who provide a number of direct social care services to older adults with LTC needs, such as assistance with personal care and domestic chores under the Korean LTCI system. The concept of turnover intention has also been defined in numerous ways [13,14,15]. This study defines it as a deliberate and conscious wish to leave an organization and an intention to seek alternative employment. This refers to both care workers leaving one care work job for another similar care work job and leaving the field altogether [14]. 

### 1.1. Aim of Study

Several studies have explored the turnover of care workers in Korea. However, most of these existing studies [9,10,16] have utilized quantitative methods, which do not sufficiently explain turnover issues from the perspectives of care workers. To fill this gap, this study used qualitative research methods to explore the reasons that care workers are likely to resign or move to other service-providing organizations. The experience of care workers in Korea has policy implications for other countries that plan to reform their LTC workforce systems to cope with ageing populations. 

### 1.2. Literature Review and Background

#### 1.2.1. The Main Factors Related to the Turnover of Care Workers

The existing literature suggests that many factors affect the turnover of care workers. This study presents the main factors, considering macro (economy), meso (organization), and micro (staff) levels. First, macro level factors of economic conditions at a country or local level, such as economic growth, unemployment rates, and comparable income, affect the turnover of care workers [17,18,19]. Some studies have found that the global financial crisis in 2008 diminished worries about the shortage of workforce [20], partly because middle-aged (50–64) care workers delayed retirement [21]. An inverse relationship between unemployment and the turnover of care workers in nursing homes has also been found. When unemployment increased, care workers were less likely to leave their roles [17]. 

Second, many existing studies have reported that organization level factors, such as job design, workload, training, and ownership of organizations, have been closely associated with the turnover of care workers, although some studies have not supported this relationship [22]. Given the prevalence of the marketization of care in many countries, it is notable that for-profit LTC organizations tend to maximize their profits by paying low wages and demanding heavy workloads, leading to the high turnover of care workers [18,23]. It has also been found that the turnover rate of personal care aides in not-for-profit organizations was lower compared to that of aides in for-profit facilities [12]. Moreover, for-profit service organizations are encouraged to recruit part-time or flexible workers under the structural circumstances of “privatization, rationalization, and increased competition” [19]. Neoliberal welfare states, such as the USA and UK, with high proportions of for-profit LTC service providers, frequently show this trend. Therefore, the working conditions of care workers are very challenging and their pay is low [3,19]. For instance, the wages of direct care workers are the lowest in the UK, and many of them receive the national minimum wage [3].

Finally, at the micro staff level, the relationships between employers, elderly clients, and care workers are a significant factor in the turnover of the workforce [16,24,25,26]. When care workers do not receive proper supervision and do not feel a connection with their employers, turnover increases. In Australia, Radford et al. [27] presented two concepts to analyze the relationships and turnover of care workers: perceived supervisor support, referring to “employees” perceptions about how much their supervisors care about their wellbeing and value their contribution to organization”, and job embeddedness, referring to “the connections and relationships employees develop over a period of time with their employer.” They found that these two concepts were very useful in predicting care workers’ intentions to stay or leave their jobs. Notably, proper treatment by supervisors motivates care workers to stay longer in their workplace [24,27]. Similarly, Bowers et al. [1] conducted in-depth interviews with care workers employed in nursing homes and found that their perceptions of being unappreciated and undervalued by the organizations for which they work influenced their turnover.

#### 1.2.2. The Development and Turnover of Care Workers in Korea 

To understand the unique characteristics and developments of care workers and the main factors that influence turnover in Korea, we need to review literature on the policy developments related to the retention of care workers. Importantly, in the past, care workers were poorly trained in the absence of a professional training and certification system. However, the systematic training of care workers has been possible in recent years through the introduction of the new LTCI and certification system. 

In doing so, the government adopted a policy of the marketization of care, which refers to government measures that allow, support, or facilitate the participation of both for-profit and not-for-profit service providers in the care market and promote the market principles of competition and choice [2,28]. This approach contributed to a rapid increase in the number of LTCI service providers because of the active participation of the for-profit sector. The Korean LTCI market has been dominated by for-profit providers, as most of the LTCI service providers were from the for-profit sector (domiciliary providers 80.9%, institutional 64.3%), while only 0.8% of domiciliary and 2.3% of institutional providers were from the public sector, and 17.7% of domiciliary and 33.2% of institutional providers were from the not-for-profit sector in 2015 [29]. Notably, the government forced many for-profit LTC service providers to establish training for care workers. Although a new certification system for care workers was introduced, the regulations regarding certification were very weak, requiring only 240 hours of training and an easy examination [7]. However, enjoying such loose regulation, many for-profit training organizations maximized their profits by producing large numbers of care workers quickly, rather than training them professionally to provide high-quality services [7]. The Korean government endeavored to increase the number of care workers to implement a new LTCI system, disregarding the quality of services. Therefore, although the number of certified care workers increased rapidly to 1,415,203 in 2016 since 2008 [30], the quality of services has been criticized repeatedly by many older adults and their family caregivers.

Many middle-aged women expected to obtain decent jobs as certified care workers. However, this belief has dwindled in the face of heavy workloads, low pay, and demanding tasks such as household chores [31]. Their working conditions are also unstable, as many care workers have been employed temporarily, such as those working at home visiting centers (86.2%) or institutional homes (25%) in 2015 [32]. Therefore, many young and middle-aged women have been reluctant to work as care workers; accordingly, the mean age of care workers is quite high in Korea. As Table 1 shows, while care workers in their forties comprise only 13.5% of all care workers, those aged 50 or over comprise more than 80% [31]. Thus, these care workers are much older compared to those in other countries, such as France, where 24% of care workers are aged 55 or over [33].

These challenges have brought about the high turnover rate of care workers in Korea. One study found that the turnover rate of nursing home care workers (62.0%) is higher than that of home care workers (48.4%) [34]. Around 60% of care workers in Seoul had turnover intentions owing to low wages, unstable job status, and demanding work [11]. Although there are too many certified care workers, as noted previously, many of them no longer want to work in the field. In 2016, 313,013 care workers provided services in the field [35]. 

Specifically, some studies found that low salaries, low job satisfaction, high job stress, high employment uncertainty, and demanding working environments influenced the turnover of care workers in Korea [11,36]. In addition, the following factors seem to significantly affect the turnover intention of care workers: self-efficacy, job identity, burnout, organizational commitments [37], income, emotional work and relationship with the elderly [16], role conflict, workload, salary level, colleague relationship [37], and abuse by the clients [36]. Due to the high turnover of care workers, many LTCI service providers have experienced a shortage of staff [10,31], and it is predicted that in 2020 and 2030, there will be a shortage of care workers by 23,882 and 111,125, respectively [38].

## 2. Methodology

### 2.1. Sampling

To explore the reasons for the high turnover of care workers from their perspectives, we adopted a qualitative research method in this study. This methodology was very useful for collecting in-depth information by gathering the vivid experiences of care workers in the field through face-to-face interviews [39,40]. In 2013, the research was conducted in two cities (Suwon-si and Yongin-si) located near Seoul in the Kyeonggi province, which contains the largest number of older people in Korea.

We conducted semi-structured in-depth interviews with 20 direct care workers. However, since one care worker (P6) did not provide meaningful comments on the research topic, she was excluded from the analysis and the remaining 19 care workers were used as the research sample. Purposive and snowballing sampling methods were used to recruit research participants who could shed light on the research topic [39]. After an interview was conducted with a care worker, they were asked to recommend other care workers who met the sampling criteria [41]: those who had worked in LTCI facilities for older adults for at least a year, knew the research topic well, and were able to communicate. Overall, interviewees were very supportive in recommending their colleagues, and most of the recommended care workers gladly participated in the interviews, although a number of care workers had difficulties in making appointments for interviews because of their work schedules.

The main characteristics of the interviewees are presented in Table 2. All of the interviewees were women, mostly in their fifties and sixties. Nine interviewees provided in-home care services and the remaining ten worked in nursing homes. Most worked in the for-profit LTC organizations and most had a long experience of providing LTCI services in the field. According to Kim et al. (2020) [42], which analyzed 1,221,085 care workers in October 2015, when categorizing them in terms of gender, the proportion of women was 93.8%, and by age, 12.1% were in their forties, 52.7% in their fifties, 30.8% in their sixties, and 1.6% in their seventies. Most of the interviewees for this study were women, with 3 people in their forties, 13 in their 50s, and 3 in their sixties. Therefore, it can be considered that the composition of age and gender was relatively representative of the population of interest. 

### 2.2. Instrument

Prior to commencing the interviews, a guide was prepared, which included questions intended to elicit the vivid experiences of care workers. These questions allowed the conversation with the care workers to be smoothly led, creating a sort of guided conversation [43]. Questions about their experience of the provision of care services in the fields and their turnover intentions were asked and probes were often used. The questions and probes used were as follows: “Would you please talk about your experiences of providing of care services for older adults?” (Probe: “How did you feel at that time?”), “Have you ever thought about quitting your job?” (Probe: If the participant said “yes”, “Can you explain in detail why you want to quit that job?”) 

### 2.3. Procedure

Semi-structured in-depth interviews were conducted in coffee shops or interviewees’ homes, wherever it was convenient for them and quiet enough to talk and record. Overall, the interviewees were very active in discussing their experiences and difficulties of working as care workers, and they reported detailed reasons for turnover intention from their viewpoints. The interviews lasted on average for 60–90 minutes. In terms of ethical considerations, there was no institutional review board at the universities in Korea at the time of this field research. Despite this, research was conducted in an ethical way. For instance, before starting the interviews, the aim and topic of the research were explained to the participants, and they were assured that they had the right to withdraw from the interview at any time and a right to request the scripts and analyzed data. All of the interviews were tape-recorded after obtaining signed informed consent forms from the interviewees. The confidentiality of the collected data and the anonymity of the research participants were explained to them. These principles were followed throughout the study.

### 2.4. Data Analysis

All recorded data were transcribed verbatim by two research assistants, who were asked to abide by the principles of confidentiality and anonymity. The transcripts were read repeatedly to become more familiar with the collected data and themes related to the research topic were sought out. To analyze the collected data, the qualitative software ATLAS-Ti version 6.2.28 (Scientific Software Development GmbH, Berlin, Germany) was used. Thematic analysis was adopted, which has been widely used by qualitative researchers [40]. First, open-coding was conducted. Rather than sticking to the existing theory and concepts in the literature, new codes were developed based on the collected data. It took a long time to complete open coding due to the large quantity of transcribed data. Axial coding was then conducted by linking the generated open codes and attempting to find the underlined themes, patterns, or concepts. Lastly, selective coding was conducted. All of the coded data, as well as the original collected data, were continually read to find core themes and check for missing data. Based on the thematic analysis, quotations that supported the main themes were found and presented.

## 3. Results

Many findings emerged from this research. In particular, four main findings relating to turnover intention were: demanding working conditions, low salary, bad relationships with directors and supervisors, and the degradation of the health of care workers.

### 3.1. Demanding Working Conditions 

Many care workers said that demanding working conditions were a key factor in their turnover intentions. First, care workers in nursing homes often said that their working conditions were very difficult, since each care worker was responsible for six to nine patients during the day and twelve to fifteen patients at night. Nighttime work is particularly stressful, since it is not easy for one care worker to meet even the basic needs of so many older adults. Moreover, to reduce personnel costs, nursing home service providers have tended to use fewer care workers. One care worker said:

*“[At our nursing home] Two care workers look after 24 elderly clients. It’s hard to work. I am so busy with work. I have no time even to write [a] daily record … Many care workers resigned [from] their jobs because the work of changing diapers was squeamish for them …”*

(P7)

Second, it was reported that many care workers in nursing homes had to work long hours, such as a double shift or working every other day. These long working hours are prevalent, often violating the Labor Act Standards in Korea [31,43]. In small nursing homes (less than 10 elderly clients), 44.1% of care workers worked every other day, and 49.2% of care workers in nursing homes (with more than nine elderly clients) worked double shifts [10].

Third, some new care workers have enormous difficulties in adjusting to the demanding tasks involved in this sort of work, such as changing elderly patients’ diapers and administering suppositories and enemas. Moreover, new care workers have difficulties in treating clients with complicated conditions, such as elderly clients with dementia who often refuse to have their diapers changed even when the diapers smell bad, and might have other behavioral and psychological symptoms that are part of their dementia. 

Finally, some home care workers reported that some older people or their family caregivers did not have a clear concept of care workers and what their role was, and mistook them for housemaids, expecting them to do all of the household chores. In other words, the working boundary of care is not limited to services such as bathing and meal assistance for elderly clients; instead, it includes cleaning homes and doing laundry. It was also reported that most supervisors of care workers did not intervene between care workers and users to tackle this issue. 

### 3.2. Low Salary 

Many care workers claimed that they were underpaid considering the difficulty of their jobs. They were in poor financial shape, and for this reason, they had entered the LTC field. However, care workers in nursing homes complained that although they worked long hours under demanding conditions, their salary was still too low.

*“The purpose of my job is to make a living. I divorced and am bad[ly] off … Yesterday, I looked after eight elderly clients and gave them bath[s] … I am exhausted and my body became swollen … But the salary payment is too low … I receive 1200 dollars per month [1 dollar = roughly 1000 Won] ... How can I live on this salary? I can’t educate my kids with this amount … It would be better to do work which can make 2000 dollars rather than this care work, 1200 dollars … So, young people don’t choose to do this care work.”*

(P15)

Because of low wages and differences in wages offered by service providers, some care workers said that they tried to move to better-paying service organizations or jobs. Given this financial motivation, some mentioned that even a slight increase in hourly payment was a reason to move to other LTCI service providers or roles. Although there were variations in salary between local areas in Korea, the monthly salary of care workers in domiciliary services and in nursing homes was on average US $949.47 (hourly US $7.28) and US $1403.81 (hourly US $8.83) in 2015, respectively [31]. Low salaries are closely associated with the price of LTCI services. The price is set annually by the long-term care committee, comprising government officials, service providers, civic organizations, and scholars. However, it has been emphasized that the price of services is still too low to pay an adequate salary to their care workers [10], even though the price has gradually increased. 

### 3.3. Bad Relationships with Directors and Supervisors

A surprising finding was that more than half of the sample reported that a bad relationship with their directors was a significant factor in moving to other organizations or leaving their jobs. Some directors looked down on their care workers and failed to consider their working situations.

*“Our directors have the mind that we had the relationship between subordinates and superiors … When I bathe the elderly clients, I am very busy and it’s a hard job for me. But, the director said to me, “Do this! Do that one!” I have only one body. How can I do this and that one together? Rather, he needs to support me. Then, I got upset and frustrated … He tells us, “Just follow my instruction. If not, write an apology and explanation!” So, care workers resign their jobs. Although we keep working here, he tells us “You leave here! I dislike you because of this and that.”*

(P14)

Such inappropriate behavior on the part of directors appears to be prevalent, even though service providers have enormous difficulties in recruiting new care workers. Direct care workers believed that some directors appeared to regard care workers as means to maximize their profits rather than colleagues to be valued and supported to provide quality services for older adults. 

In addition, some home care workers reported that they also had poor relationships with their supervisors, such as social workers and nurses, due to unfair administrative burdens. Supervisors allocate cases and working schedules, and these issues are a source of conflict. In particular, it was found that case allocation is a very sensitive issue for home care workers, since it is directly related to their workload and hours. Home care workers receive salary based on their working hours rather than fixed salaries in Korea; therefore, case allocation is directly linked to their income. From the perspectives of care workers, unfair case allocation or working schedules created severe conflict between them and their supervisors, which leads to turnover among care workers.

*“The relationship with [a] supervisor is important. When I am in charge of two cases, if one case is gone, I have only one case [to be] in charge of. The reason why we are working here is to make money and so one case is not enough to do so … When the care worker has a bad relationship with the supervisor, the care worker has to wait longer since other care worker[s] can receive the new case earlier than I …”*

(P18)

Overall, the findings of this study suggest that some Korean care workers are under-appreciated and under-valued by their directors, and that administrative workers are not fairly treated by their supervisors. Some care workers had poor working relationships with their directors or supervisors and did not have a sense of belonging to the organization [27], which prompted their intention to leave or move.

### 3.4. Deterioration of Care Workers’ Health

A final reason for turnover is the deterioration of care workers’ health. Many interviewees reported that the tasks care workers have to perform often lead to musculoskeletal diseases, making them unable to work. Many care workers reported that excessive or demanding body care services, such as bathing, moving heavy male patients and changing positions was difficult for them. One said:

*“It’s inevitable for me to leave this job … The government seems to leave the situation of turnover as it is … Since we are looking after the frail elderly patients, not healthy people, our energy is getting drained. My body and spirit are exhausted. It is obviously physically challenging … It’s really nonsense to carry on my job here receiving the small salary.”*

(P9)

As presented in Table 2, care workers in the study worked between 1 and 11 years, with most caregivers working for over 5 years. Long years of continuous care work appear to damage the overall health and well-being of care workers. Moreover, care workers in the LTC sector are more likely to experience physical injuries, back pain, and exhaustion compared to other service sectors, and they are more likely to be exposed to violence and threats while receiving limited support from directors or supervisors [44].

Given that the average age of care workers in the study was 54 years, caring for older people was unlikely to be easy for them. Moreover, the challenging situations that Korean care workers face of poor working conditions found in this research also negatively affects the health of care workers, which leads to turnover.

## 4. Discussion

The study conducted semi-structured interviews with 19 care workers in the Korean LTCI and found several reasons for turnover intention of care workers. Since this is small-scale qualitative research, we need to be careful not to generalize the findings of the research, which were, firstly, that care workers face a number of demanding working conditions such as heavy caseloads, long working hours, demanding physical work, and inappropriate requests. In particular, care workers in nursing homes are more likely to find their working conditions very challenging, since each care worker has to care for twelve to fifteen elderly clients at night and has to work long hours, such as double shifts. This finding is consistent with previous studies, which have shown that around half of care workers in nursing homes worked double shifts in 2012 [10], 72.4% of them worked 40–45 hours per week, and 21.3% of them worked over 45 hours per week in 2015 [31]. Moreover, some new care workers had enormous difficulties in adjusting to demanding care work and treating elderly clients with complex needs, such as those with severe dementia. 

Secondly, in terms of the low salary of care workers, this finding is consistent with previous literature [10,11]. The interviewees reported that they became care workers for financial reasons; however, since their salary was too low and the work demanding, they tended to move to better-paying organizations. Care workers are sensitive to financial issues, since a previous study [43] reported that about half of care workers sampled in Seoul were main breadwinners. However, since the LTCI system was initially designed and operated in a conservative way under the principle of cost containments [5], Korean governments have endeavored to tightly control LTCI budgets by setting a low price for LTCI services. Therefore, fundamentally, it is difficult to pay care workers adequately. Service providers and care workers have asked the government for an increase in pay every year. However, since the prices of services are closely associated with the financing and sustainability of the LTCI system, governments have very carefully and slowly increased the prices of LTCI. Given the financial needs and motivation of care workers, an increased salary is one of the best ways to reduce turnover [44]. Accordingly, the government started to provide additional payments (maximum US $100 per month) to care workers who work more than 40 hours per week in 2013. Although this might help some care workers who meet the criteria, the overall salary of care workers is still low.

Thirdly, this study found that bad relationships between care workers and their directors or supervisors are a major reason for turnover intentions. Surprisingly, some directors gave orders unilaterally, without considering their working situations; some directors even openly detested care workers. Similarly, some care workers reported that their supervisors completed administrative works, such as case allocation and working schedule, unfairly, which became a source of conflict and led to turnover of care workers. Care workers believed that some service providers view care workers as means to maximize their profit. This is also related to the low social status of care workers in Korea [31]. However, it should be noted that the frequent changes of staff among care workers negatively affects the profits of service providers as a result of costs associated with recruiting and training new staff [2,12].

Finally, regarding the degradation of care workers’ health, some of the sample suffered from musculoskeletal problems as a direct result of long years of providing demanding services, such as excessive body care for elderly clients. Since health is the outcome of a number of social and economic factors, other factors of turnover intention of care workers already mentioned in this research might affect care workers’ health negatively. Moreover, given the fact that those aged 50 or above made up more than 80 percent of the care workers in Korea as noted [31], they would be vulnerable to a decline of health as a result of long years of care and demanding working conditions.

## 5. Conclusions

The findings of this research appear to suggest that the turnover of care workers is related to a number of policies and practices under the Korean LTCI system. Although the data was gathered in 2013 and a number of new supporting policies for care workers, such as supplementary pay, have been implemented since then, the structural challenges of care workers have remained. 

Most of all, the government should scrutinize care workers’ working practices. In particular, since long working hours often violate the Labor Act Standards, such inappropriate practices should be explored in depth, and appropriate practices that keep the law and consider the situations of care workers should be implemented. Moreover, the training system should be revised because 240 hours of training time to become a certified care worker is too little. As Kim et al. (2020) [42] shows, an increase in training hours can offer an opportunity to improve the overall capacity of care workers. In particular, by providing training on how to form appropriate relationships with service users, colleagues, and supervisors, the turnover rate of care workers as a result of bad relationships with their supervisors would decrease. 

Secondly, since the government sets low prices for LTCI services, the salary of care workers is fundamentally restricted. Therefore, the prices need to be increased to the level at which the salary of care workers is equivalent to other similar types of occupations, such as medical assistants. However, the sustainability of LTCI financing should also be considered, including raising the cost of insurance and tax [45]. Thirdly, the inappropriate behavior of service directors and supervisors needs to be changed through training when the service directors and supervisors open their organizations or start to work, such that they treat care workers as valuable and respected colleagues. Finally, occupational health and safety insurance needs to cover care workers who have suffered from musculoskeletal conditions. Currently, insurance considers such issues to be a symptom of ageing rather than a consequence of care work, and thus the insurance does not cover it [30].

In terms of the limitations of the study, this research was very small-scale and explored only the perspectives of care workers; therefore, diverse perspectives of stakeholders, such as directors and supervisors, should be explored in future large-scale studies. We suggest that larger-scale, mixed research should be carried out, exploring the issues of turnover of care workers in terms of health, relationships, payments, and rewards. 

## Figures and Tables

**Table 1:** The proportion of age of care workers according to the types of services in Oct. 2015 [31].

Types	Total	10s	20s	30s	40s	50s	60s	70s
Domiciliary services	427,206	9 (0.0%)	1314 (0.3%)	8628 (2.0%)	57,580 (13.5%)	183,750 (43.0%)	149,313 (35.0%)	26,612 (6.2%)
Institutional services	56,703	3 (0.0%)	481 (0.8%)	1107 (2.0%)	6851 (12.1%)	29,899 (52.7%)	17,452 (30.8%)	910 (1.6%)

**Table 2:** The main characteristics of interview participants.

Participants	Age	Kind of Service	Ownership of Provider	Years of Experience
P1	65	in-home	FPO *	6
P2	57	nursing	FPO	5
P3	46	in-home	FPO	4
P4	57	nursing	FPO	3.75
P5	56	nursing	FPO	1
P7	50	nursing	FPO	5
P8	51	in-home	NPO **	10
P9	50	in-home	FPO	7.5
P10	52	in-home	FPO	6
P11	65	in-home	NPO	15
P12	53	in-home	FPO	4
P13	62	in-home	NPO	11
P14	58	nursing	FPO	7
P15	57	nursing	FPO	4.5
P16	52	nursing	NPO	4
P17	48	nursing	FPO	2.6
P18	44	in-home	NPO	5
P19	58	nursing	FPO	4.8
P20	52	nursing	FPO	7

* FPO = For-profit organization. ** NPO = Not-for-profit organization.
